# Feature tracking strain is similar to harmonic phase cardiac magnetic resonance in Fontan patients: a validation study

**DOI:** 10.1186/1532-429X-16-S1-P106

**Published:** 2014-01-16

**Authors:** Shafkat Anwar, Elisha J Fogel, Ravi Doddasomayajula, Alexander Davidson, Marc S Keller, Matthew A Harris, Kevin K Whitehead, Mark A Fogel

**Affiliations:** 1 Pediatrics, Cardiology, The Children's Hospital of Philadelphia, Philadelphia, Pennsylvania, USA; 2 Pediatrics, Radiology, The Children's Hospital of Philadelphia, Philadelphia, Pennsylvania, USA

## Background

Feature tracking strain (FTS) is a new technique to evaluate myocardial deformation from routinely acquired cardiac magnetic resonance (CMR) cine images, however, it has not been validated in single ventricle patients. The purpose of this study is to validate FTS against myocardial tagged harmonic phase (HARP) images, considered the reference standard in non-invasive deformation analysis.

## Methods

We retrospectively analyzed CMRs of 15 consecutive single ventricle Fontan patients (Table 1) with myocardial tagging and balanced steady state free precession (SSFP) cine images in the same study. Off-line analysis was performed on basal, mid and apical short axis levels using Diagnasoft Virtue (v4.5.1) and TomTec 2D Cardiac Performance Analysis (v 1.0) for HARP and FTS, respectively. Lagrangian endocardial strain was compared between techniques.

**Table 1:** Demographics.

Subjects, n	15
Age in years, media (range)	18 (5-38)

Single ventricle morphology (LV:RV)	7:8

Double outlet right ventricle, n	5

Hypolastic left heart syndrome, n	4

Tricuspid atresia	3

Pulmonary atresia, intact ventricular septum, n	1

Unbalanced AV canal, n	1 unbalanced to left 1 unbalanced to right

## Results

**Figure 1:**
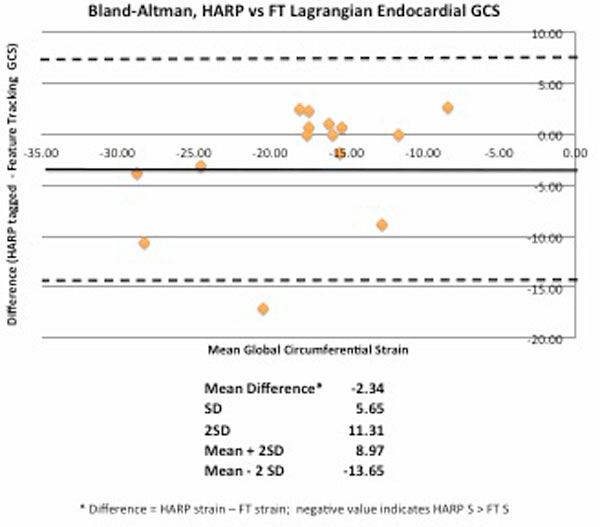
**Bland-Altman HARP vs Feature Tracking Strain**.

There was good correlation in global circumferential strain (GCS) between HARP and FTS, Pearson r = 0.67, p = 0.006. Average GCS was -19 (95% CI -4.3 to -33.8) from HARP and -16.7 (95% CI -6.4 to -26.9) from FTS. FTS yielded lower values (bias -2.3) than HARP (Figure 1), equivalent to a bias of 12.3% (average bias of FTS/average HARP × 100). This difference was not significant via Student's T-test (t = 3, DF 13, p = 0.1). Analysis of variance (ANOVA) showed considerable overlap in measurements, F = 2.64, p = 0.04. Intraclass correlation was 0.58, indicating moderate agreement.

## Conclusions

Feature tracking analysis has moderate agreement with grid-tagged HARP measurements of circumferential strain in Fontan patients, with a trend towards lower strain values via FTS. Further validation of FTS in a large sample is warranted.

## Funding

Dr Kevin K. Whitehead: NIH K23 Grant HL089647 from the National Heart, Lung and Blood Institute. Dr. Mark Fogel: NIH R01HL098252-01, "Understanding mechanisms of Fontan failure and key predictors for patient outcome."

